# Magic Doping and Robust Superconductivity in Monolayer FeSe on Titanates

**DOI:** 10.1002/advs.202003454

**Published:** 2021-02-14

**Authors:** Tao Jia, Zhuoyu Chen, Slavko N. Rebec, Makoto Hashimoto, Donghui Lu, Thomas P. Devereaux, Dung‐Hai Lee, Robert G. Moore, Zhi‐Xun Shen

**Affiliations:** ^1^ Stanford Institute for Materials and Energy Sciences SLAC National Accelerator Laboratory Menlo Park CA 94025 USA; ^2^ Departments of Physics, Applied Physics, and Materials Science and Engineering Geballe Laboratory for Advanced Materials Stanford University Stanford CA 94305 USA; ^3^ Stanford Synchrotron Radiation Lightsource SLAC National Accelerator Laboratory Menlo Park CA 94025 USA; ^4^ Department of Physics University of California at Berkeley Berkeley CA 94720 USA; ^5^ Materials Sciences Division Lawrence Berkeley National Laboratory Berkeley CA 94720 USA; ^6^ Materials Science and Technology Division Oak Ridge National Laboratory Oak Ridge TN 37831 USA

**Keywords:** FeSe, heterostructures, interfacial charge transfer, magic doping, superconductors

## Abstract

The enhanced superconductivity in monolayer FeSe on titanates opens a fascinating pathway toward the rational design of high‐temperature superconductors. Utilizing the state‐of‐the‐art oxide plus chalcogenide molecular beam epitaxy systems *in situ* connected to a synchrotron angle‐resolved photoemission spectroscope, epitaxial LaTiO_3_ layers with varied atomic thicknesses are inserted between monolayer FeSe and SrTiO_3_, for systematic modulation of interfacial chemical potential. With the dramatic increase of electron accumulation at the LaTiO_3_/SrTiO_3_ surface, providing a substantial surge of work function mismatch across the FeSe/oxide interface, the charge transfer and the superconducting gap in the monolayer FeSe are found to remain markedly robust. This unexpected finding indicate the existence of an intrinsically anchored “magic” doping within the monolayer FeSe systems.

## Graphical abstract

Monolayer FeSe/LaTiO_3_/SrTiO_3_ heterostructures with varied LaTiO_3_ thicknesses are synthesized by molecular beam epitaxy in‐situ connected to a synchrotron angle‐resolved photoemission spectroscope. The electron accumulation at the LaTiO_3_/SrTiO_3_ surface provides a substantial surge of work‐function mismatch across the FeSe/oxide interface, but the charge transfer and superconducting gap of FeSe remain remarkably robust, indicating an intrinsically anchored “magic” doping in FeSe.

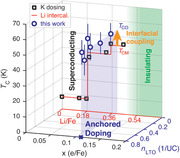

The quest for raising superconducting transition temperature (*T*_C_) has been a central theme of material science research.^[^ ^1^ ^]^ A remarkable triumph is the monolayer FeSe grown on SrTiO_3_ (noted hereafter as 1UC FeSe/STO, UC standing for unit cell), in which superconductivity is significantly enhanced compared to its bulk form.^[^ ^2^, ^3^, ^4^ ^]^ Experimental evidence so far suggests that the source of elevated *T*_C_ is twofold: extra electron doping and interfacial mode coupling.^[^ ^5^, ^6^, ^7^, ^8^ ^]^ The role of interfacial coupling effect has been extensively discussed in the literature.^[^ ^5^, ^7^, ^9^, ^10^, ^11^, ^12^, ^13^, ^14^ ^]^

To study the doping effect, researchers have employed alkaline metal (Li, Na, K, and Cs) atom adsorption for bulk and multilayer FeSe. Due to the low ionization energy, alkali metal dosing or intercalation acts as a charge injector. Doping level can be tuned and phase diagrams of *T*_C_ are obtained.^[^ ^8^, ^15^, ^16^, ^17^, ^18^ ^]^ Comparing these phase diagrams, two important pieces of information can be drawn. First, there exists a superconductor–insulator transition in a higher doping regime.^[^ ^8^, ^18^ ^]^ Remarkably, transport measurements with Li intercalation exhibit apparent phase‐separation features across this transition, indicative of a first‐order phase transition instead of a continuous one. Second, a sharp discreteness of possible *T*_C_’s is found in the continuous tuning of Li doping, and the discreteness is smoothed with additional disorders, such as replacing Fe with Cu, S with Se, or increasing the size of dopant atoms (e.g., from Li to Na). Although the spatially average carrier concentration is fixed by such doping method, in the presence of disorder, the carrier density distribution is often inhomogeneous. These observations call for a doping method for FeSe without the introduction of impurity dopants.

Monolayer FeSe grown on STO substrate is doped by interfacial charge transfer.^[^ ^12^, ^19^ ^]^ Interestingly, the majority of reported high‐quality monolayer FeSe films grown on different types of perovskite titanate substrates, including STO(100),^[^ ^5^, ^19^ ^]^ STO(110),^[^ ^13^ ^]^ and BaTiO_3_^[^ ^9^ ^]^ with varied dielectric and work function properties, exhibit similar levels of doping (≈ 0.10–0.12 electrons per Fe atom). For increasing the doping ability of the substrate, the LaTiO_3_ (LTO)/STO heterostructure is an excellent candidate. LTO provides Ti^3+^ and forms a 2D electron gas (2DEG) accumulated at the surface of the LTO/STO heterostructure,^[^ ^20^, ^21^, ^22^ ^]^ indicating a lowered surface work function. When growing 1UC FeSe on top of LTO/STO, we would be able to provide an additional chemical potential difference across the interface for charge transfer to FeSe from LTO/STO, while maintaining other properties similar to 1UC FeSe/STO without introducing additional disorders due to the structural similarity between STO and LTO. Different from testing different perovskite substrates for FeSe arbitrarily, we can precisely control the thickness for LTO and thus provide the essential systematics. The *in situ* synchrotron angle‐resolved photoemission spectroscope (ARPES) can unambiguously determine the doping of FeSe by Fermi surface volume. Avoiding the use of Li or other alkaline atom adsorption, we can rule out any possibility of Li ordering. Importantly, the insertion of LTO systematically controls the interfacial work function difference for charge transfer, which is thermodynamically distinct from alkaline metal dosing or intercalation, where the number of electrons injected is proportional to the number of adsorption atom number.

In this work, we systematically synthesize 1UC FeSe films on LTO/STO heterostructures in two separate but *in situ* connected molecular beam epitaxy (MBE) chambers, then examine the low‐temperature electronic structure of the grown films by*in situ* ARPES in Stanford synchrotron radiation lightsource (SSRL). By varying LTO thickness, we find that the itinerant electron density at the surface of the LTO/STO heterostructure surges to more than 4 × 10^14^ cm^−2^, but surprisingly neither the doping nor the superconducting gap of 1UC FeSe film grown on it exhibit noticeable changes. Our results show that the superconductivity in 1UC FeSe thin films is robust and accompanied with an anchored “magic” doping level.

We grow LTO films on STO substrates after the growth of STO buffer layers in a shutter‐controlled oxide MBE chamber, monitored by *in situ* reflective high energy electron diffraction (RHEED). The STO buffer layer is grown using a shuttered approach for deposition of different elements.^[^ ^23^, ^24^, ^25^ ^]^ To grow LTO, we use a shuttered approach with on‐the‐fly adjustment of the shutter times layer‐by‐layer to maximize the RHEED intensity oscillations. We then transfer the LTO films *in situ* to a separate chalcogenide MBE chamber for the growth of 1UC FeSe. After vacuum post‐annealing, the samples are transferred *in situ* to the ARPES chamber at SSRL beamline 5‐2 for measurement. We control the annealing conditions identical for different samples to avoid annealing‐related variation.^[^ ^26^, ^27^ ^]^ More details of growth and measurement conditions can be found in Supporting Information.

The ARPES spectra of a 1UC FeSe/5UC LTO/STO heterostructure sample are shown in **Figure** **1** as a representative example. It has a Fermi surface with only electron pockets near M point (Brillouin zone corner), and a Luttinger volume count that gives 0.11 ± 0.01 electrons per Fe atom (Figure 1b). The top of the hole bands at *Γ* are ≈75 meV below Fermi level, and the bottom of electron bands at M is about 55 meV below Fermi level (Figure 1a,c). Replicas of electron and hole bands are also visible in the spectra at M point, which is clearer after taking second energy derivative of the image (Figure 1d). At low temperature, there is a superconducting gap of 14 meV with clear back bending, as is shown in the energy distribution curves (EDCs) in Figure 1e. The gap basically disappears at 57 K or above (Figure 1f). The band structure features of 1UC FeSe on 5UC LTO film resemble that of 1UC FeSe/STO, which is unexpected considering the additional electrons provided by La in the LTO/STO.

**Figure 1:**
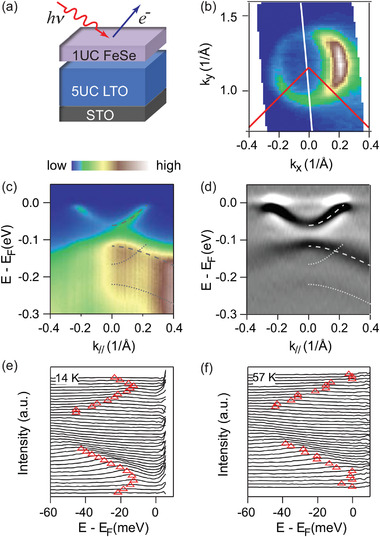
ARPES characterizations of 1UC FeSe/5UC LTO/STO films. a) Schematic diagram of the material structure. b) The Fermi surfaces of electron pockets near the zone corner M. Red lines indicate the Brillouin zone edges. c,d) Spectra and its second derivative taken at zone corner M, along the cut shown with the white line in (b). The dashed and dotted curves are guides to the eye for the main bands and the replica bands. e,f) EDCs for the spectra taken at the zone corner M. The EDCs are divided by Fermi distribution function at measurement temperatures 14 and 57 K, respectively. Red triangles indicate the energies of maximum intensities within *E* − *E*_F_ = [−50 meV, 0]. All spectra here are taken with 28 eV photons.

To probe this artificial structure systematically, we measure 1UC FeSe grown on LTO films with different thickness, as shown in **Figure** **2**. All the 1UC FeSe films have almost identical doping level regardless of whether it is on STO substrate or any thickness of LTO films (Figure 2a–d). In sharp contrast, the electronic structure changes dramatically from STO to LTO with different thickness prior to the FeSe deposition (Figure 2e–h). The STO substrate (preannealed with the same condition for later FeSe growth) shows clear Fermi surfaces, consistent with previous studies.^[^ ^28^, ^29^ ^]^ The three largest Fermi surfaces consist of one circular d*_xy_* subband, one horizontally elongated oval d*_yz_* subband, and one vertically elongated oval d*_xz_* subband. There exist Fermi surfaces originated from higher‐order subbands, but since they are much smaller and the electrons occupying these subbands are much farther away from the surface, these higher order subbands are less relevant to our focus. When we grow 1UC LTO on top of STO, all the three Fermi surfaces become significantly larger.^[^ ^22^ ^]^ As the number of LTO layers increase, more electrons are provided by La, giving rise to even larger Fermi surfaces. For the 3UC and 20UC cases shown in Figure 2g,h, the edges of the Fermi surfaces of the d*_yz_* and d*_xz_* subbands extends beyond the Brillouin zone. This observed surge of accumulated electrons at the surface of the LTO/STO structure is a result of the deepened confinement potential well at the LTO/STO surface, indicative of a decreased surface work function. Note that minor portion of measured electrons are possibly associated with oxygen vacancies induced in a double Auger process with photon exposure to 84 eV (higher than 38 eV) photons until saturation,^[^ ^30^, ^31^ ^]^ yet the electrons measured in LTO/STO exceed that of STO substrate by far even before such exposure to high‐energy photons, as shown in Supporting Information.

**Figure 2:**
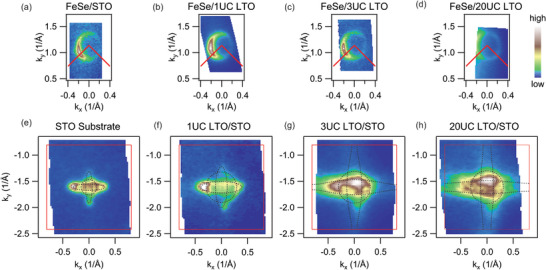
Systematic Fermi surface maps of FeSe and LTO/STO heterostructures. a–d) The Fermi surface maps near M for 1UC FeSe films on STO substrate, 1UC, 3UC, and 20UC LTO films, respectively, taken with photon energies between 25 and 28 eV. e–h) The Fermi surface maps of STO substrate, 1UC, 3UC, and 20UC LTO films grown on STO, respectively, taken with photon of 84 eV and circular right polarization. Red lines indicate the Brillouin zones. Dashed lines are guides to the eye of the three outermost Fermi surfaces. To estimate the Fermi surface sizes, both maps with circular right and linear vertical (Figures S3 and S5, Supporting Information) polarizations are used.

We summarize our results in **Figure** **3**. Figure 3a shows the density of the accumulated electrons at the surface of LTO/STO with different LTO thickness. We count the lowest d*_xy_*, d*_yz_*, and d*_xz_* bands in the ARPES Fermi surface maps (higher‐order subbands are much smaller and the electrons are located much deeper away from the interface, thus they are much less relevant to the interfacial effects we focus on). Electron density quickly increases as the LTO film thickness changes from 0UC (bare STO) to 3UC, and reaches a plateau of ≈5 × 10^14^ cm^−2^ for 3UC and thicker LTO films. The saturation behavior is consistent with numerical simulation in Supporting Information. Electron density observed from the surface‐sensitive ARPES for thinner LTO films are lower, due to the electron redistribution between LTO and the STO layers. LTO films have effectively lower work functions, thus certain amount of itinerant electrons will be transferred to the STO layers. When LTO films are thicker, the interface between LTO and STO, where the charge transfer occurs, becomes deeper and less influential to the surface, resulting in an increased and saturated observable electron density. Based on the simulation in the Supporting Information, with the dense electron accumulation for LTO thicker than 3UC, the surface work function of the LTO/STO is lowered by ≈ 0.7 V.

**Figure 3:**
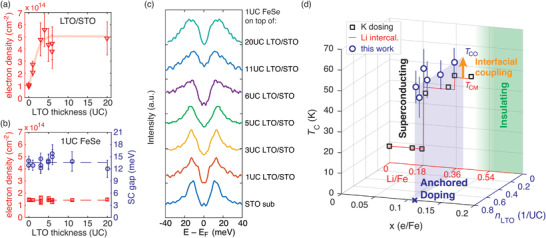
a) ARPES measured electron density of LTO films as a function of film thickness. The red thick curve is a guide to the eye for the electron density trend of increase and saturation. b) (left) Electron density of 1UC FeSe on LTO/STO heterostructures with different LTO thickness, in red squares. (right) Superconducting gap at temperatures below 20 K for 1UC FeSe on LTO/STO heterostructures with different LTO thickness, in blue circles. Dashed lines show the average values for all samples. c) Representative symmetrized EDCs at *k*_F_ with measurement temperatures lower than 20 K for different thickness of LTO insertion. d) The blue open circles show the *T*_C_ and doping in the unit of electron per Fe atom of 1UC FeSe/LTO/STO with various LTO/STO electron concentration *n*_LTO_, in the unit of electron per in‐plane LTO/STO unit cell, as measured by ARPES Fermi surface maps. *T*_C_ values are converted using superconducting gap data, using a coefficient 2*∆*_0_/*k*_B_ *T*_C_ = 5.7, consistent with literature.^[^ ^11^ ^]^ The transparent blue vertical plane corresponds to the average values of doping and *T*_C_, denoted as *T*_CO_, in which “O” stands for oxide substrates. The black squares are *T*_C_ data extracted from Figure 4 in ^8^] (a K dosed FeSe ARPES experiment), as a function of electron doping *x* (e/Fe) measured by ARPES Fermi surface maps. The red curve is a reproduction of discrete *T*_C_ steps for lithium ionic solid gated FeSe thin flakes, as a function of nominal Li content with Li/Fe ratio adapted from Figure 5 of ^18^]. Note that the content (Li/Fe) axis is rescaled to match the actual electron doping axis *x* (e/Fe) as measured by ARPES Fermi surface maps. *T*_CM_ represents the maximum *T*_C_ recorded in doped bulk/multilayer FeSe systems. The insulating regime indicated by the green shaded area represents the findings from both references.^[^ ^8^, ^18^ ^]^

In sharp contrast, the electron density as measured by ARPES Fermi surface maps and superconducting gap at low temperature for 1UC FeSe/LTO/STO films remain nearly unchanged with different LTO thickness, as plotted in Figure 3b. For 0UC (STO bare substrate), the 1UC FeSe film on top exhibits higher doping than the STO substrate prior to deposition of FeSe. Starting from 1UC LTO, the electron density on the surface of LTO/STO is larger than that of the 1UC FeSe grown on top. For the cases where the LTO thickness is 3UC and above, the electron density of LTO/STO is about three times that of the 1UC FeSe. Regardless of the dramatic changes of the electron density in the LTO/STO substrate, both electron density and superconducting gap of 1UC FeSe remain basically the same within experimental errors. The doping of 1UC FeSe/LTO/STO falls largely between 0.10 and 0.12 electrons per Fe atom, or between 1.31 × 10^14^ and 1.58 × 10^14^ cm^−2^, and the gap is mostly between 12 to 16 meV.

As the doping level is anchored for 1UC FeSe despite large changes in the substrate, an immediate implication could be that the doping is from the monolayer FeSe film itself alone, such as Se vacancies.^[^ ^32^, ^33^ ^]^ Yet, this requires a blockage of electron tunneling between FeSe and the oxide substrate so that the work function across the interface does not need to be balanced. This is highly unlikely since it contradicts direct experimental evidence of charge transfer.^[^ ^12^, ^34^ ^]^ As electrons move across the interface between FeSe and the titanate substrate, an energy equilibrium state would be established by the redistribution of electrons. Here, we increase the electron density in titanate substrates to ≈ 5 × 10^14^ cm^−2^, much larger than the typical electron density of monolayer FeSe/STO, not only effectively creating metal–metal contact, but also greatly lowered the effective work function of the substrate surface. Note that the nature of metal‐metal contact is insensitive to interface details such as terminations. After FeSe is grown on LTO/STO, for balancing additional work function difference across the interface, FeSe electron density would be expected to be much higher than the case on STO substrates (see more details in Supporting Information). Therefore, the ARPES measured unchanged doping of 1UC FeSe/LTO/STO points to some unusual intrinsic properties of FeSe.

Similar phenomenon of robust superconductivity is also seen in the Li‐intercalated FeSe thin flake experiment,^[^ ^18^ ^]^ where discrete *T*_C_ changes are observed as Li is continuously intercalated (see Figure 3c). After the system reaches and plateaus at the highest *T*_C_ (≈ 44 K), further doping brings the system into an insulator with marked discreteness in transport measurements. The superconductor–insulator phase transition is also observed in K dosing experiments on multilayer FeSe thin films.^[^ ^8^ ^]^ The doping for maximal *T*_C_ (≈ 45 K) is ≈ 0.11 electrons/Fe atom, similar to the case of 1UC FeSe on STO or LTO. Above this doping, the system gradually transitions into an insulating phase. The major difference between the Li and K experiments is the level of discreetness. The K experiment seems to be a smoothed version of the Li experiment. In our work, we find an unusually anchored doping level despite strong interface electron accumulation as a “clean” doping channel, since we do not introduce extra disorder by ad‐atoms or vacancies. Moreover, as a method of varying interfacial work function difference rather than direct injection of electrons (as in Li or K experiments), by LTO insertion we have not observed obvious signatures of the insulating phase nor phase separation. These results are summarized in Figure 3c, where we combine our observations on monolayer FeSe/LTO/STO, in which the doping is anchored at 0.11 ± 0.01, with the Li and K experiments.^[^ ^8^, ^18^ ^]^

Our results of a “magic” anchored doping on 1UC FeSe despite large variation in substrate carrier density, combined with the studies listed above, show that the doping of FeSe is far from being fully understood. Below we propose a scenario that could possibly explain the unique phenomena of FeSe doping levels. In theory, there might exists a first‐order phase transition between a superconducting phase with a maximum possible doping of ≈ 0.11 and an insulating phase at a higher doping governed by a yet concealed order for FeSe. In real material, true first‐order phase transition may not exist with the presence of disorder, but by approaching the “clean” limit, first‐order‐like behaviors, such as phase separations, could be observed. Thus, electron injection higher than ≈ 0.11 by Li dosing to FeSe could have formed phase separations between the superconducting phase and the insulating phase.^[^ ^18^ ^]^ However, in the FeSe/LTO/STO system, the extra work function difference, which would facilitate higher electron transfer at the interface in a trivial case, need to exceed a critical potential barrier originated from the theoretical first‐order phase transition to transit into the insulating phase. Our observation of an anchored doping and absence of the insulating features suggests such critical potential barrier is still higher than the increased work function difference built by LTO insertion. Even if small amount of insulating phase would exist and form phase separations due to finite temperature, only the ≈ 0.11 doping superconducting phase would be visible by ARPES, and the minor portion of insulating phase would be hard to discern by APRES due to the low intensity and the defuse nature of the spectra.^[^ ^8^ ^]^ This scenario might also explain why most reported high‐quality monolayer FeSe films grown on different types of perovskite titanate substrates^[^ ^5^, ^9^, ^13^, ^19^ ^]^ “magically” exhibit similar ≈ 0.11 doping in ARPES, which coincidentally corresponds to the maximum *T*_C_ found in doped multilayer systems.^[^ ^8^, ^17^ ^]^ In the cases of K/Cs dosing or excess Se, additional disorders are introduced and the discreteness is smoothed, making the transitions more continuous and less first‐order‐like.^[^ ^35^ ^]^ This leads to a continuous change in Fermi surface volume and *T*_C_.^[^ ^6^, ^8^, ^17^, ^36^, ^37^ ^]^ Interestingly, the *T*_C_ evolution with Na intercalation represents an intermediately smoothed case between Li and K.^[^ ^18^ ^]^

Another aspect of our results is the largely invariant superconducting gap under great change of itinerant electron density in the substrate. For the interfacial electron–phonon coupling with phonon modes in directions parallel to the surface, dense itinerant 2DEG on LTO surface can provide strong screening effect. However, because the in‐plane motion of carriers in the substrate cannot screen the charge transfer induced electric field that is perpendicular to the interface, we expect the extra carriers in LTO cannot screen the long‐wavelength longitudinal optical phonon modes associated with ionic vibrations that are also perpendicular to the interface and modulating the interfacial electric field. It is precisely this type of phonon which is suggested to enhance the superconductivity in 1UC FeSe/STO system.^[^ ^5^, ^38^, ^39^ ^]^ Therefore, the largely unchanged superconducting gap of FeSe on LTO with different thicknesses suggests that the relevant interfacial coupling at FeSe/oxide interface are strongly selective for the phonon modes involved.

In conclusion, empowered by the *in situ* oxide and chalcogenide MBE systems that are directly coupled to the synchrotron ARPES, we have systematically studied the electronic structure 1UC FeSe on LTO/STO heterostructures. We find that the doping level and enhanced superconductivity of the monolayer FeSe is exceptionally robust in spite of substantial increase of electron density in the substrate. The indicated anchored “magic” doping level suggests a unique underlying material property, posing a challenging target for theoretical and computational materials science research.

## Conflict of Interest

The authors declare no conflict of interest.

## Supporting information

Supporting Information

## Data Availability

The data that support the findings of this study are available from the corresponding author upon reasonable request.
