# Supplementary material for: Magic Doping and Robust Superconductivity in Monolayer FeSe on Titanates
Source: Adv Sci (Weinh). 2021 Feb 14;8(9):2003454. doi: 10.1002/advs.202003454 (PMC8097367; doi:10.1002/advs.202003454)
Supplement: Supplementary file 1 — Supporting Information [file ADVS-8-2003454-s001.pdf]

© 2021 Wiley-VCH GmbH

ADVANCED SCIENCE
Open Access

Supporting Information

for *Adv. Sci.,* DOI: 10.1002/advs.202003454

Magic Doping and Robust Superconductivity in Monolayer FeSe on Titanates

Tao Jia, Zhuoyu Chen*, Slavko N. Rebec, Makoto Hashimoto, Donghui Lu, Thomas P. Devereaux, Dung-Hai Lee, Robert G. Moore, and Zhi-Xun Shen*

Supporting Information

Magic Doping and Robust Superconductivity in Monolayer FeSe on Titanates

Tao Jia, Zhuoyu Chen, Slavko N. Rebec, Makoto Hashimoto, Donghui Lu, Thomas P. Devereaux, Dung-Hai Lee, Robert G. Moore, and Zhi-Xun Shen**

Section 1. Synthesis of FeSe/LTO/LTO heterostructures and ARPES measurements

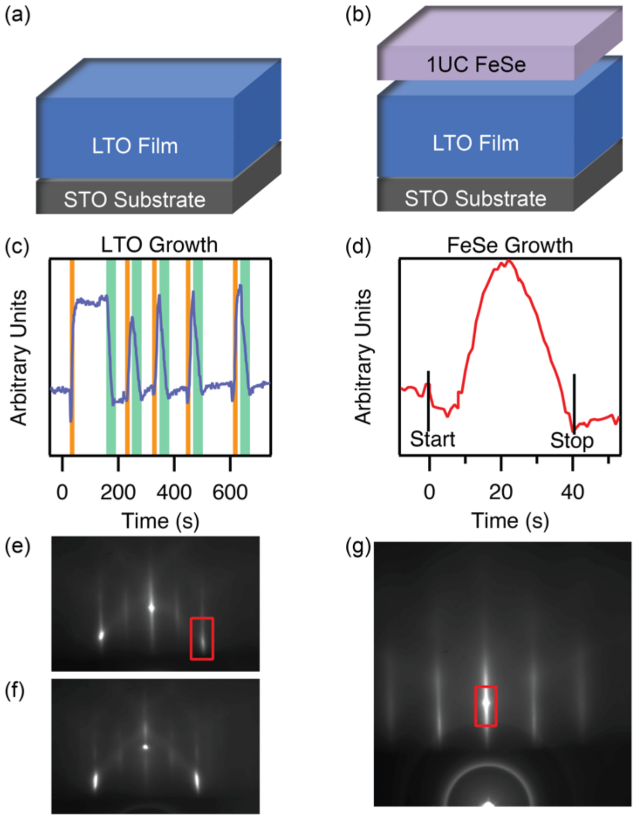

Figure S1. Sample growth. (a-b) Schematic diagrams of LTO/STO and 1UC FeSe/LTO/STO heterostructures. (c) The RHEED oscillations of a shutter-controlled 5UC LTO film growth as an example. The vertical axis is the integrated intensity within the red window in (e). Orange and green colored backgrounds indicate the time windows when the La and Ti shutters are opened, respectively. White backgrounds correspond to pauses during the growth. (d) The RHEED oscillations of a 1UC FeSe film on the grown 5UC LTO film shown in (c) and (e). The vertical axis is the integrated intensity within the red window in (g). (e) The RHEED pattern after the growth along the STO [100] direction of a 5UC LTO film. (f) The RHEED pattern after the growth along the STO [100] direction of another 5UC LTO film, for which the shutter time is adjusted from layer to layer. (g) The RHEED pattern after the growth along the STO [100] direction of a monolayer FeSe film on the 5UC LTO film shown in (e).

All samples are grown on 0.05% Nb Doped STEP STO purchased from Shinkosa. The substrates are mounted to inconel sample holders using silver paste. The samples are first exposed to 6×10^{-6} Torr oxygen partial pressure and heated to 900 °C for substrate annealing for 30 minutes before the LTO growth at 820 °C in the same oxygen environment. The RHEED is aligned along the STO [110] direction during growth. Before an LTO growth, an STO buffer layer of 5-20 unit cells (UC) is first grown on the STO substrate to ensure the surface smoothness and consistency. Three cells are used for growth: a differentially pumped source loaded with ultra high purity Sr (99.95%), a high temperature cell loaded with ultra high purity Ti (99.995%), and a high temperature cell loaded with ultra high purity La (99.995%). The source flux and deposition rates are calibrated and set using a quartz crystal microbalance.

Growth is done using a shuttered approach. A typical LTO (STO) recipe starts with the La (Sr) shutter opening and ends with the Ti. Before growth, we usually align the tilting angle of the STO substrate to maximize the central streak intensity.[1] The STO buffer layer is grown using a shuttered approach for deposition of different elements.[2–4] To grow LTO, we use the shuttered approach with on-the-fly adjustment of the shutter times layer-by-layer to maximize the RHEED intensity oscillations. Namely, we don't seek to keep the shutter time exactly consistent from layer to layer for both La and Ti, but with a general shutter time guideline that is found from previous test growths. We majorly focus on maximizing the RHEED quality during the growth. Focusing on the (01) streak intensity oscillation as shown in **Figure S1e**, if we saw a "double-peak" feature (similar to what is described in ref.[2]) in the previous RHEED oscillation, we typically decreased the La shutter time by shutting the shutter slightly before the RHEED reached peak intensity. If we saw an obviously lower peak intensity, we typically increased the La shutter time so that The RHEED intensity went higher. If we saw a lower valley intensity than general, we decreased Ti shutter time by shutting the shutter slightly before the RHEED reached typical valley intensity. If we saw a higher valley intensity than general, we increased Ti shutter time by shutting the shutter only when the RHEED reached typical valley intensity. By this adjustment strategy, the RHEED oscillations generally converge to a largely consistent amplitude. This strategy brings about a shutter time variation. Among samples that we have measured in ARPES (15 films), the standard deviation for La shutter time is 14% (change from average shutter time for each layer) and standard deviation for Ti is 8%. We discuss the effect of the shutter time variation below. Different from the case shown in Figure S1c in which each layer having exact same shutter time, Figure S1f corresponds to a film of same 5UC LTO thickness but with the first layer having ∼ 20% shorter and second layer having ∼ 50% longer La shutter time compared to the average. This longer shutter time is compensated by shorter shutter time in latter layers. This variation in shutter time could be one source of cation disorder since it would require redistribution of cations within ∼ 1 unit cell thickness. We find that, at the relatively high growth temperature (820 °C) we use, the effect of shutter time variation is not obvious in the final RHEED patterns, as can be seen in the comparison between Figure S1e and S1f, indicating the cation disorder level on the surface is lower than what RHEED can resolve. This is consistent with the finding that shown in ref.[4], in which we intentionally change the growth so the oscillations got "flipped" in phase, but after the oscillations got "flipped" back, a good final oscillation can still be obtained and ARPES still shows a surface electron accumulation after UV radiation. We speculate that the surface sticking coefficient gradually changes from STO buffer layer to thicker and thicker LTO films, but further experiments and simulations are needed to clarify. After closing each shutter, the growth is usually paused for RHEED intensity to saturate. After saturation, longer pause does not have an additional noticeable effect on the growth of the film. Post growth the samples are cooled down in oxygen background and then transferred in situ to chalcogenide MBE chamber or ARPES chamber for further growth or measurement.

FeSe growths are done in a chalcogenide MBE chamber, at a base pressure of 8×10^{-11} torr. The LTO/STO films are transferred *in situ* to the chalcogenide chamber and heated up to 360 °C for growth without additional annealing. Ultra-high purity iron (99.995%) and selenium (99.999%) were then deposited onto the substrate. The film was then annealed at 500 °C for 2 hours. The growth conditions of FeSe on STO (001) are described in ref.[5], except that after growth they are annealed at 500 °C for 2 hours for consistency. Figure S1d and S1g shows the RHEED oscillation during growth and the resulting RHEED pattern, respectively.

After growth, the films are transferred in situ to the ARPES end station of the Stanford Synchrotron Radiation Lightsource beamline 5-2. The base pressure in the ARPES chamber is lower than 4×10^{-11} Torr. The measurements on FeSe are done with photon energy of 25-28 eV, and the best energy resolution for gap measurement is ~ 6 meV. The measurements on LTO films and STO substrates are done using photon energy of 84 eV, and the energy resolution is better than 31 meV. The angular resolution is better than 0.1°.

Section 2. ARPES spectra of 1UC FeSe/LTO/STO and LTO/STO

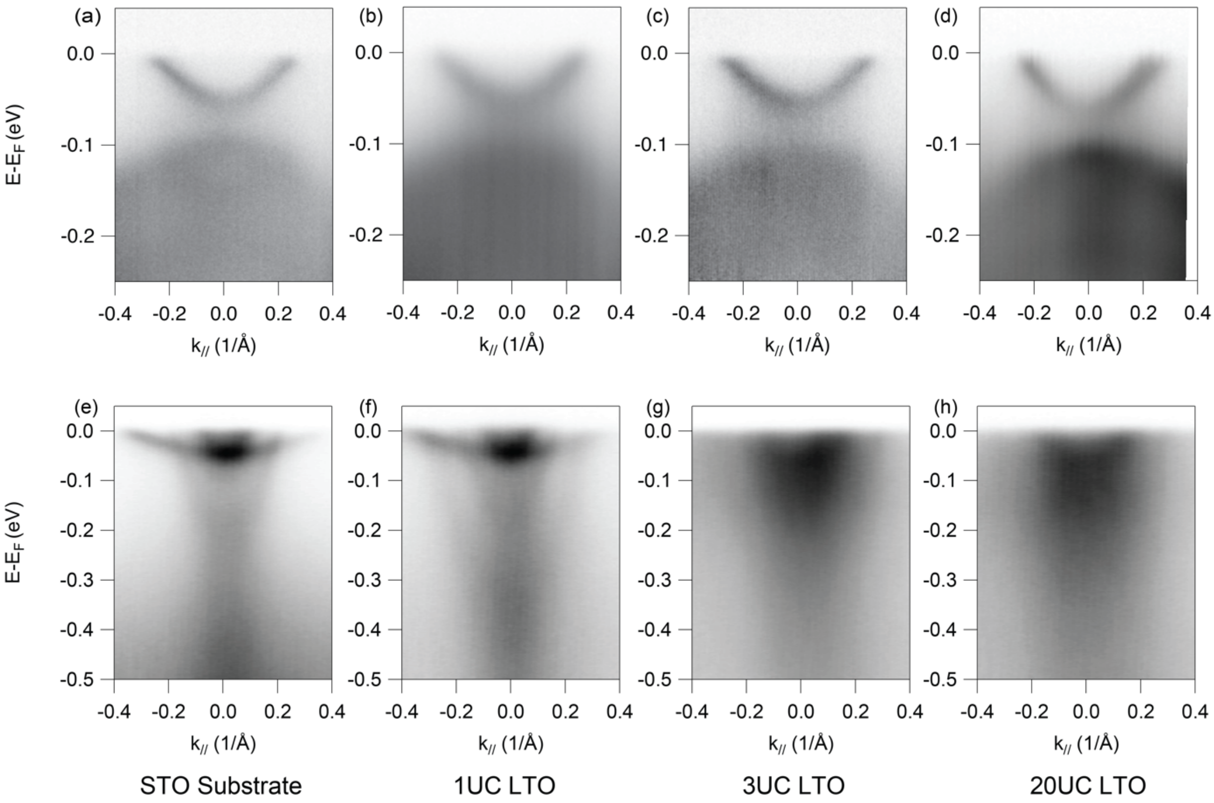

Figure S2. ARPES spectra of FeSe/LTO/STO and LTO/STO films with different thickness along high symmetry cuts. (a-d) ARPES spectra of 1UC FeSe/LTO/STO heterostructure with different LTO thickness near zone corner along the direction from zone center to zone corner. The number of LTO layers are 0 (STO substrate), 1, 3, 20, respectively. (e-h) ARPES spectra of LTO/STO heterostructure at the center of the second Brillouin zone $(0, -2\pi/a)$, where a is the lattice constant of the heterostructure. The number of LTO layers are 0 (STO substrate), 1, 3, 20, respectively.

Section 3. Polarization dependence for LTO/STO ARPES

The Fermi surface map of LTO films displayed in Figure 2 in main text is measured with circular right (CR) polarization. **Figure S3** (e-h) shows that the elliptical pocket along the direction of k_y is missing if linear vertical (LV) polarization is used. This is consistent with previous research on STO 2DEG.[6]

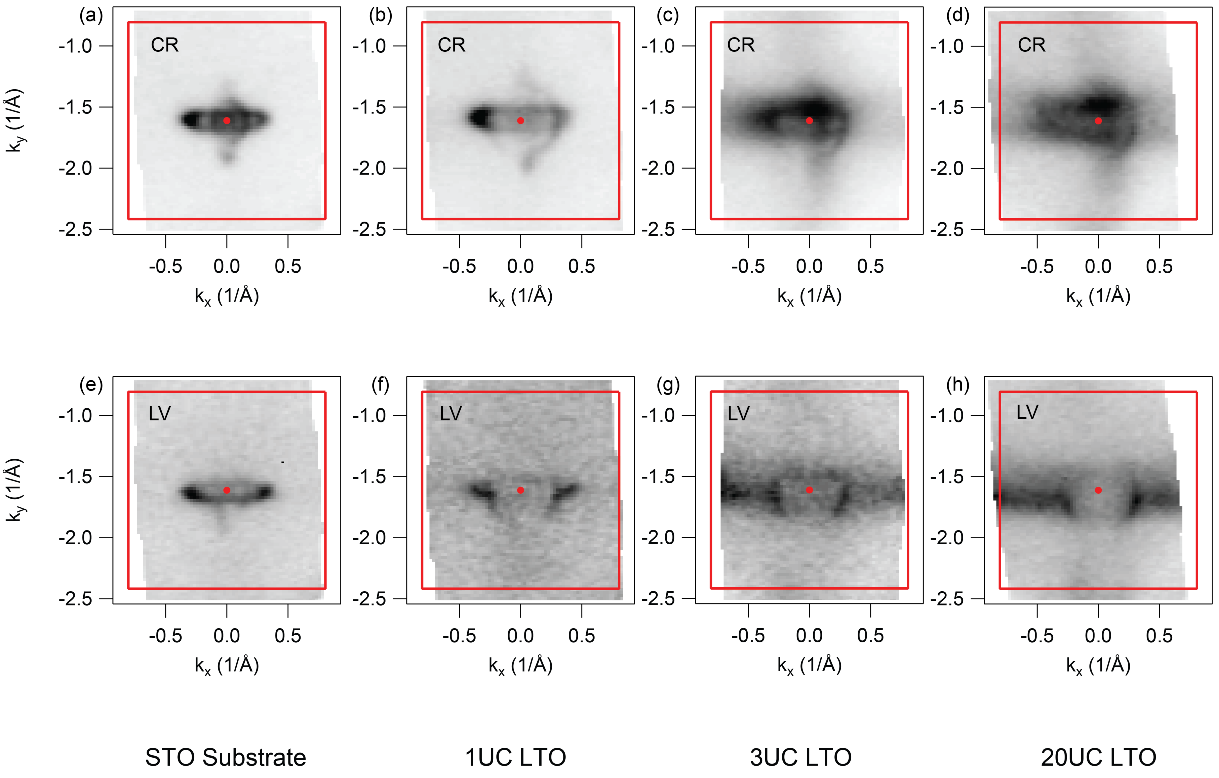

Figure S3. (a-d) Fermi surface map of LTO/STO heterostructure with different LTO thickness taken with circular right (CR) polarization. These are the same figures as Figure 2 (e-h) in main text. The number of LTO layers are 0 (STO substrate), 1, 3, 20, respectively. (e-h) Fermi surface map taken at the same setting as (a-d), except with linear vertical (LV) polarization.

Section 4. ARPES spectra before exposure to high-energy photons

2DEGs can emerge on STO surface after exposure to high-energy photons. This process, as is discussed in the main text, is achieved by oxygen vacancies created in a double-Auger process.[7, 8] However, in order to acquire high-quality spectra of 2DEGs on LTO/STO, one has to measure the centers of higher Brillouin zones, thus a higher photon energy than the Auger process threshold (38 eV) is necessary. In our research, most data on LTO/STO are taken with 84 eV photons. Note that the double Auger effect is a relatively minor effect compared to the amount of electron provided by LTO. Here we discuss this effect for completeness.

To avoid the photon-induced electrons, we measure the first Brillouin zone (Γ(0, 0)) using 28 eV photons. Although the Fermi surfaces are only partially visible at Γ(0,0), likely due to matrix element effects, important conclusions can still be drawn. From **Figure S4**, we can see that without radiation of 84 eV photons the pockets are bigger for 1UC LTO compared to that of STO, and even bigger for 5UC LTO. This demonstrates that there are intrinsic electrons without the exposure to higher energy photons. It also supports our findings in Figure 2 and 3 in the main text that the electron density increases rapidly as LTO thickness increases from 0UC (STO) to 1UC to more than 3UC. Furthermore, this is consistent with our understanding that photon-induced electrons are created in a parallel channel (from oxygen vacancies) to the intrinsic electrons of LTO (from La-donated electrons), thus the electrons from the two processes should be largely additive.

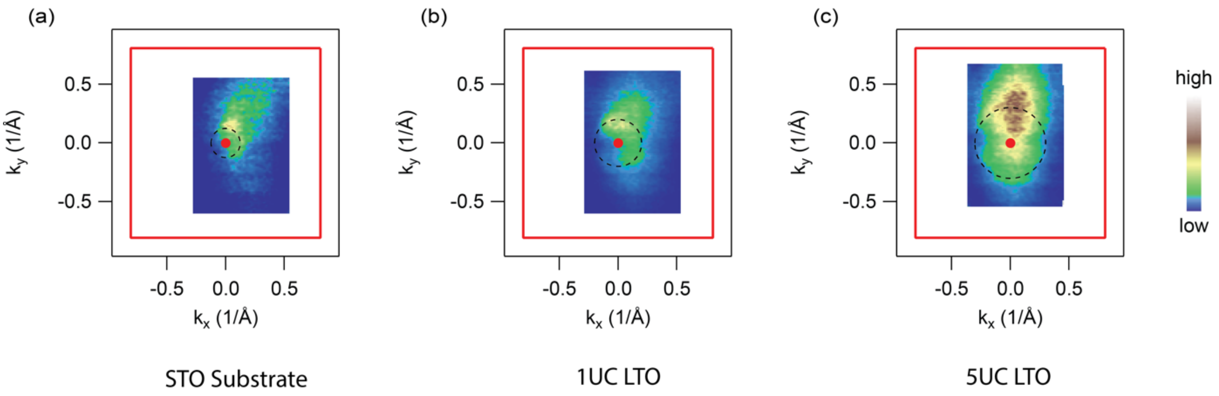

Figure S4. LTO/STO heterostructures measured with 28 eV photons. (a) intensities near EF for STO substrates without exposure to 84 eV photons. (b-c) same as top left, but for 1UC and 5UC LTO films on STO substrates, respectively.

Section 5. Additional data of LTO/STO 2DEG

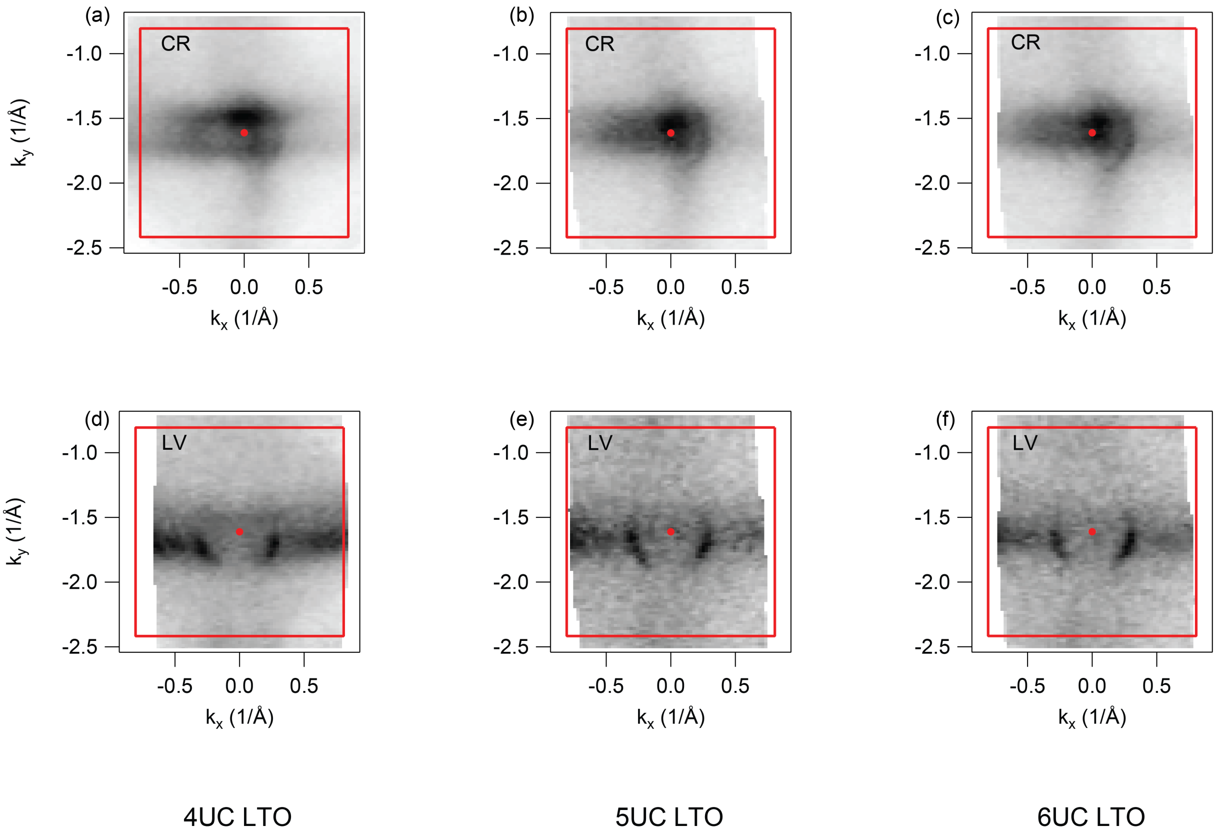

Figure S5. (a-c) Fermi surface maps of 4, 5 and 6UC LTO films measured with circular right (CR) polarized photons. (d-f) Fermi surface maps of 4, 5 and 6UC LTO films measured with linear vertical (LV) polarized photons.

Section 6. Temperature and LTO thickness dependence of superconducting gap for 1UC FeSe/LTO/STO

The temperature dependent superconducting gap was extracted from the energy distribution curves at k_F symmetrized at E_F for multiple samples, yielding similar T_C as 1UC FeSe/STO, which is consistent with the gap size at lowest temperatures. Figure S6 (a-d) show the temperature dependence of superconducting gap for a 1UC FeSe/5UC LTO/STO samples and a 1UC FeSe/11UC LTO/STO sample. The gap values as a function of temperature are fitted using the formula $\Delta(T) = \Delta_0 \tanh(k\,(T_C/T - 1))$. Figure S6 (e) shows the symmetrized EDCs for lowest temperature gap (< 20 K) of FeSe samples with different LTO thickness.

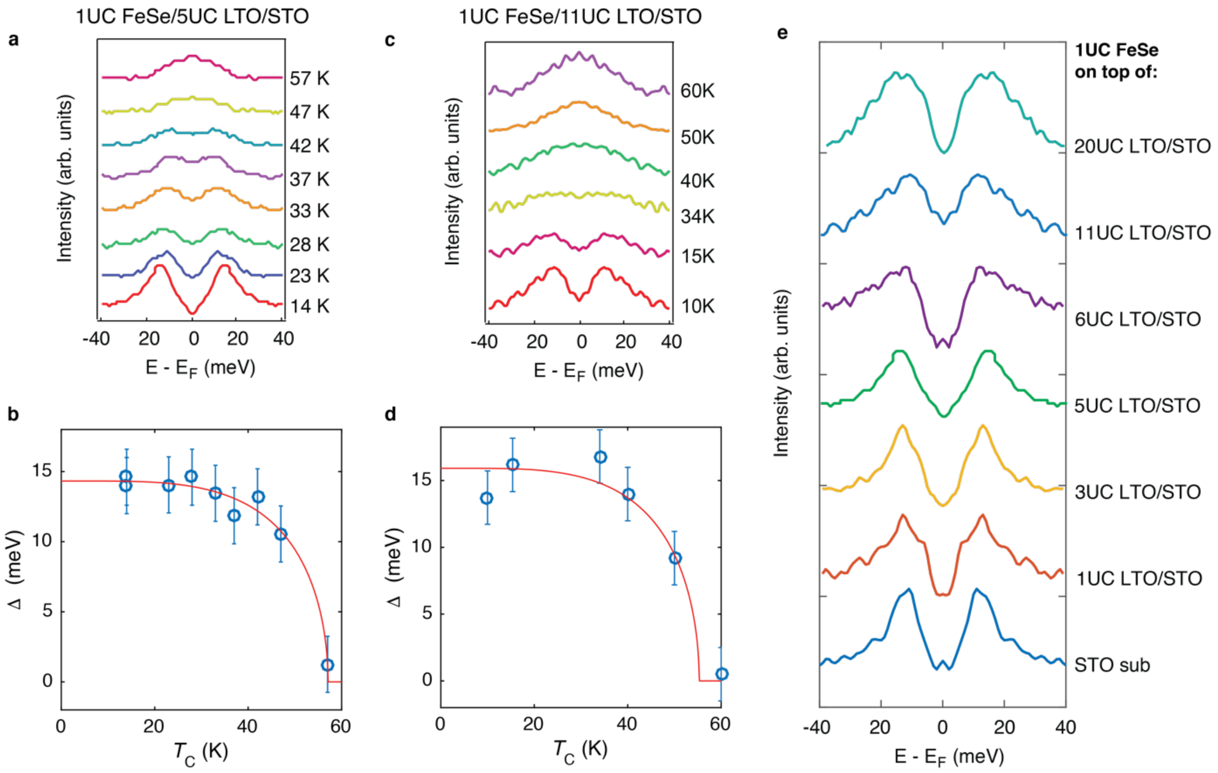

Figure S6. Temperature dependence of superconducting gap for a 1UC FeSe/5UC LTO/STO sample (a,b) and a 1UC FeSe/11UC LTO/STO sample (c,d). (a,c) Energy distribution curves (EDCs) at k_F at different temperatures, symmetrized at $E = E_F$. (b,d) Temperature dependence of superconducting gap. Blue open circles are measured gap values extracted using the model in ref.[9], and red curve is a mean-field fitting. The fitting of the gaps yields Δ_0 = 14 meV, T_C = 57 K for (b), and Δ_0 = 16 meV, T_C = 55 K for (d). (e) Symmetrized EDC at k_F with temperature lower than 20 K for samples with different LTO thickness. Same figure as Figure 3 (c) in the main text.

Section 7. Simulation of monolayer FeSe/LTO/STO

The simulation aims to calculate the supposed charge transfer amount for monolayer FeSe/LTO/STO heterostructure based on work-function balancing, without considering first-order phase transitions and insulating phase due to correlations. This simulation is based on an assumption that there is no hybridization between FeSe and LTO or STO electronic orbitals, which is partly justified by the ARPES measurement of monolayer FeSe/LTO/STO that shows basically identical band structure as doped multilayer/bulk FeSe. In particular, a tight-binding plus Poisson equation self-consistent calculation is used for the LTO/STO heterostructure to simulate the band bending and band structure. Electron correlation in LTO is not considered, thus the simulation is more accurate in the thin limit of LTO. This simulation is not intended to provide quantitative conclusion, but to clarify qualitative behaviors of the charge transfer across the FeSe and LTO/STO interface.

List of notations

e : electron charge
ε_0 : electrostatic constant of vacuum.
ε_r : effective dielectric constant between FeSe & oxide.
n_t: 2D density of transferred electron to FeSe.
$E = (n_t\, e)/\varepsilon_r\, \varepsilon_0$: electric field between FeSe and oxide.
$V = E\, d$: electric potential difference.
d : distance between FeSe and oxide.
$W_{FeSe}\,(n_t)$: FeSe work function as a function of n_t. $W_{LTO}\,(n_t)$: LTO work function at the surface.
c : coefficient for FeSe work function shift with doping.
t & t' : hopping energy for Ti 3d orbitals.
k_x, k_y, k_z : electron momenta in LTO/STO.
n_{2D} : total 2D electron density in all LTO/STO layers. n_{La} : 2D electron density donated by La in one LTO layer. l : number of LTO layers in the LTO/STO structure.
r : the ratio of La atoms that donates electron.
ε_{STO} : dielectric constant of STO.
ε_{inf} : dielectric constant of STO at infinite electric field. ε_{zero} : dielectric constant of STO at zero electric field.

General description

Figure S7 schematizes the energy diagram for FeSe and LTO/STO heterostructure before and after contact. Before contact, since the additional LTO layers on top of STO serves as the electron donor to the system, electrons accumulate at the top surface and form a band bending, resulting in a lowered work function $W_{LTO}\,(n_t)$ (< 4.5 V, the work function of STO). When FeSe contacts with the LTO/STO, charge transfer occurs. Namely, LTO/STO loses electrons and FeSe gains electrons, which should make W_{LTO} larger and W_{FeSe} smaller. The quantitative amount of change of work function is associated with the amount of transferred electron n_t. With extra electrons, the FeSe monolayer is negatively charged. The created electric field builds a potential drop V between the FeSe and the outmost oxide layer. As a consequence of the charge transfer, the chemical potential across the interface is balanced, giving rise to the following equation.

$$V\,(n_t) = W_{FeSe}\,(n_t) - W_{LTO}\,(n_t) \qquad (1)$$

in which all three terms are functions of nt. By solving this equation, we can obtain the amount of transferred charge. In the following sections, each of these three terms will be discussed in details.

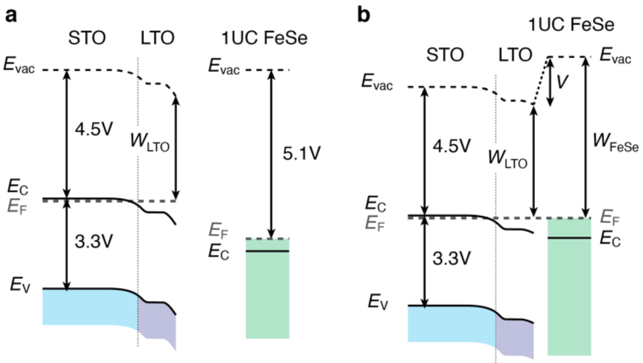

Figure S7. Schematics for FeSe and LTO/STO heterostructure chemical potential alignment before (a) and after (b) contact.

Potential difference between FeSe and oxide $V(n_t)$

It can be derived that $V(n_t) = n_t\, e\, d/(\varepsilon_r\, \varepsilon_0)$, in which d = 8Å is the spacing between FeSe and oxide, obtained from STEM literature,[10, 11] and ε_r is the effective dielectric constant between FeSe and oxide. ε_r is an important parameter, since it sets the capacitance value for the interface capacitor. We obtain this value based on experimental measurements of the FeSe/STO interface charge transfer.[12] Specifically, FeSe/STO has a charge transfer of $n_t \sim 0.11$ e/Fe; the STO band bending upward is measured to be 0.1 V; STO work function is 4.5 V; and the work function of FeSe on STO is measured to be 5.0 V. Thus, V = 5.0 V − 4.5 V − 0.1 V = 0.4 V, and ε_r can be back calculated to be 52.

LTO/STO surface work function $W_{LTO}(n_t)$

The accurate solution of the band bending at the surface and interface of this heterostructure requires a self- consistent method incorporating both band calculation and the spatially dependent electrostatics. In particular, the dielectric constant of STO is highly nonlinear and electric field dependent, spanning from more than 10^4 to as low as ~ 10. Electric field forms a confinement potential quantum well at the surface of the LTO/STO heterostructure, and electrons in the 3d orbitals forms multiple subbands, or referred as quantum well states.

We integrate the Poisson equation for electrostatics and a tight-binding model for quantum mechanics to simulate the electron distribution of LTO/STO heterostructure. This model is adapted from reference.[13] Itinerant electrons occupy d_{xy}, d_{yz}, and d_{xz} orbitals, in which electrons travel with a heavier mass in one direction and a lighter mass in the other two directions. The in-plane degree of freedom (spanned by x and y directions) has the simple solution shown below.

$$E_{\text{in-plane}} = \begin{pmatrix} -2t\cos k_x - 2t\cos k_y & 0 & 0 \\ 0 & -2t'\cos k_x - 2t\cos k_y & 0 \\ 0 & 0 & -2t\cos k_x - 2t'\cos k_y \end{pmatrix} \quad \begin{Bmatrix} xy \\ yz \\ xz \end{Bmatrix}$$

in which t and t' are the hopping energy for electrons moving in the light and heavy directions, respectively. The values of these parameters are determined by fitting ARPES measured band structures. We find t = 497 meV, and t' = 28 meV.

In the out-of-plane z direction, we solve it self-consistently considering both the tight-binding solution of electron distribution within the confinement potential quantum well and the specific shape of the

quantum well which is calculated based on the electron distribution using Poisson equation (or Gauss's Law).

In particular, we look for eigenstates and eigenvalues of the sum of the hopping matrix and the electric potential matrix.

$$H_{dxy} + U = \begin{pmatrix} 0 & t' & 0 & 0 & \cdots \\ t' & 0 & t' & 0 & \cdots \\ 0 & t' & 0 & t' & \cdots \\ 0 & 0 & t' & 0 & \cdots \\ \vdots & \vdots & \vdots & \vdots & \ddots \end{pmatrix} + \begin{pmatrix} u_1 & 0 & 0 & 0 & \cdots \\ 0 & u_2 & 0 & 0 & \cdots \\ 0 & 0 & u_3 & 0 & \cdots \\ 0 & 0 & 0 & u_4 & \cdots \\ \vdots & \vdots & \vdots & \vdots & \ddots \end{pmatrix}$$

$$H_{dyz} + U = \begin{pmatrix} 0 & t & 0 & 0 & \cdots \\ t & 0 & t & 0 & \cdots \\ 0 & t & 0 & t & \cdots \\ 0 & 0 & t & 0 & \cdots \\ \vdots & \vdots & \vdots & \vdots & \ddots \end{pmatrix} + \begin{pmatrix} u_1 & 0 & 0 & 0 & \cdots \\ 0 & u_2 & 0 & 0 & \cdots \\ 0 & 0 & u_3 & 0 & \cdots \\ 0 & 0 & 0 & u_4 & \cdots \\ \vdots & \vdots & \vdots & \vdots & \ddots \end{pmatrix}$$

$$H_{dxz} + U = \begin{pmatrix} 0 & t & 0 & 0 & \cdots \\ t & 0 & t & 0 & \cdots \\ 0 & t & 0 & t & \cdots \\ 0 & 0 & t & 0 & \cdots \\ \vdots & \vdots & \vdots & \vdots & \ddots \end{pmatrix} + \begin{pmatrix} u_1 & 0 & 0 & 0 & \cdots \\ 0 & u_2 & 0 & 0 & \cdots \\ 0 & 0 & u_3 & 0 & \cdots \\ 0 & 0 & 0 & u_4 & \cdots \\ \vdots & \vdots & \vdots & \vdots & \ddots \end{pmatrix}$$

in which u_i is the electric potential at the ith unit cell counting from the outmost surface of the LTO/STO heterostructure. With a specific known quantum well (i.e. known u_i), we are able to obtain the eigenstates and eigenvalues. Considering electric potential energy, kinetic energies from all three x, y, and z directions, E_F is determined by the total 2D electron density n_{2D} (a function of E_F). $n_{2D} = l\, n_{La} - n_t$, in which l is the number of total LTO layers, and n_{La} is the electron density donated by La in one single LTO layer. $n_{La} = r \times 6.6 \times 10^{18}$, in which r is the ratio of La atoms that actually donate electrons. With known eigenstates, eigenenergies and E_F, we can further calculate the electron distribution in each unit cell in the LTO/STO heterostructure. On the other hand, with the known electron distribution, we calculate the electric potential (i.e. u_i) based on Poisson equation incorporating the electric field dependent dielectric constant of STO $\varepsilon_{STO} = \varepsilon_{inf} + \varepsilon_{zero} /(1+E/E_C)$, in which ε_{STO} is the dielectric constant function, ε_{inf} is the dielectric constant when electric field is infinite, ε_{zero} is the dielectric constant at zero field, E is the electric field, and E_C is a constant. We fit the 10 K data from ref.[14] to obtain the constants: $\varepsilon_{zero} = 20700$, and $E_C = 1 \times 10^5$V/m.

Results and Discussions

We solve the problem $V(n_t) = W_{FeSe}(n_t) - W_{LTO}(n_t)$. **Figure S8** shows the simulation results of LTO/STO electron distribution and band bending before and after contact with FeSe, in which Figure S8a & S8e are results based on reference[12] for comparison. As can be seen, for all thicknesses of LTO, the band bending is significantly decreased after charge transfer, but the accumulated electrons at the interface is not depleted. We also find that the transferred electrons are mostly originally located at the first two unit cells before contact. **Figure S9** shows the amount of transferred charge to FeSe n_t simulated in the model as a function of LTO thickness. As can be seen, n_t surges rapidly for the first and second LTO layers, then mostly saturates thereafter, with an ultimate doping that is about twice as large as the 0UC (bare STO) case.

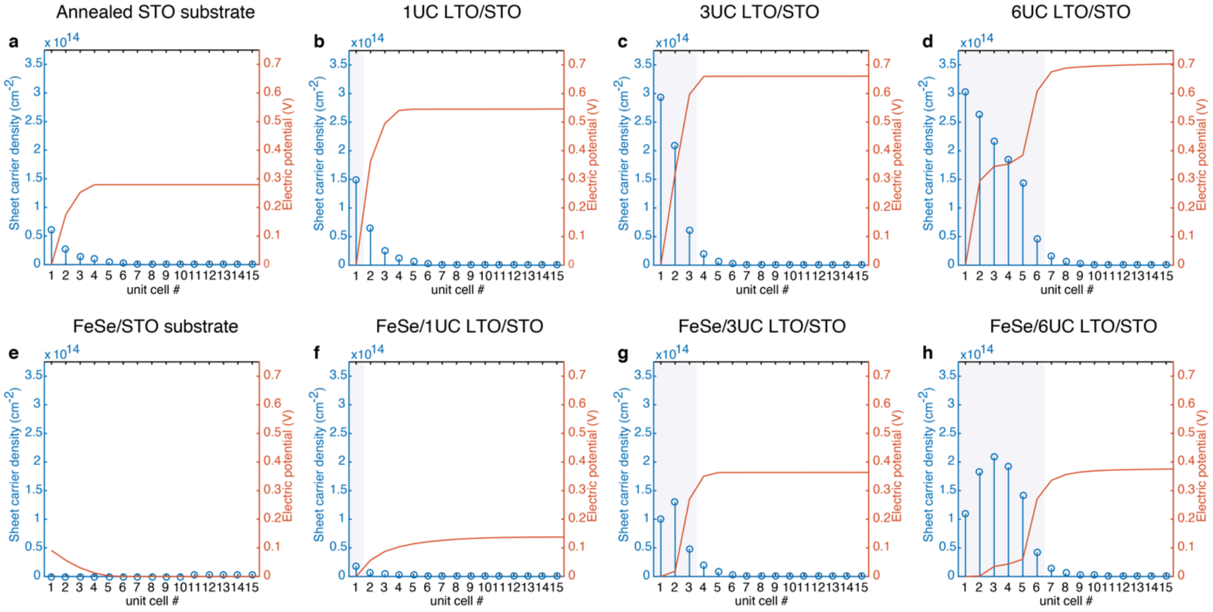

Figure S8. Electron distribution and band bending in the LTO/STO heterostructure simulated with the described model. (a) & (e) are results based on reference.[12] (a-d) are cases before contact with FeSe. (e-h) are cases after contact with FeSe. Blue shaded regions indicate the position of LTO layers.

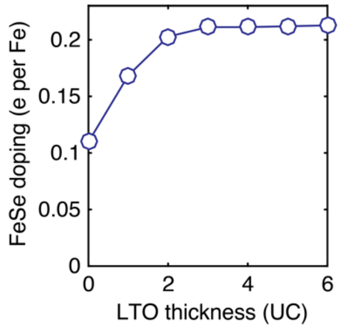

Figure S9. transferred charge to FeSe n_t simulated in the model as a function of LTO thickness.

Supporting Information References

[1] H. Y. Sun, Z. W. Mao, T. W. Zhang, L. Han, T. T. Zhang, X. B. Cai, X. Guo, Y. F. Li, Y. P. Zang, W. Guo, J. H. Song, D. X. Ji, C. Y. Gu, C. Tang, Z. B. Gu, N. Wang, Y. Zhu, D. G. Schlom, Y. F. Nie, X. Q. Pan, Nat. Commun. **2018**, *9*, 2965 (2018).

[2] J. Haeni, C. Theis, and D. Schlom, Journal of Electroceramics **2000**, *4*, 385.

[3] C. M. Brooks, L. F. Kourkoutis, T. Heeg, J. Schubert, D. A. Muller, and D. G. Schlom, Appl. Phys. Lett. **2009**, *94*, 162905.

[4] S. N. Rebec, T. Jia, H. M. Sohail, M. Hashimoto, D. Lu, Z.-X. Shen, and R. G. Moore, PNAS **2019**, *116*, 16687.

[5] J. J. Lee, F. T. Schmitt, R. G. Moore, S. Johnston, Y.-T. Cui, W. Li, M. Yi, Z. K. Liu, M. Hashimoto, Y. Zhang, D. H. Lu, T. P. Devereaux, D.-H. Lee, Z.-X. Shen, Nature **2014**, *515*, 245.

[6] A. F. Santander-Syro, O. Copie, T. Kondo, F. Fortuna, S. Pailhès, R. Weht, X. G. Qiu, F. Bertran, A. Nicolaou, A. Taleb-Ibrahimi, P. Le Fèvre, G. Herranz, M. Bibes, N. Reyren, Y. Apertet, P. Lecoeur, A. Barthélémy, M. J. Rozenberg, Nature **2011**, *469*, 189.

[7] M. Knotek, P. J. Feibelman, Phys. Rev. Lett. **1978**, *40*, 964.

[8] S. M. Walker, F. Y. Bruno, Z. Wang, A. de la Torre, S. Riccó, A. Tamai, T. K. Kim, M. Hoesch, M. Shi, M. S. Bahramy, P. D. C. King, F. Baumberger, Adv. Mater. **2015**, *27*, 3894.

[9] M. Norman, M. Randeria, H. Ding, J. Campuzano, Phys. Rev. B **1998**, *57*, R11093.

[10] F. Li, Q. Zhang, C. Tang, C. Liu, J. Shi, C. Nie, G. Zhou, Z. Li, W. Zhang, C.-L. Song, K. He, S. Ji, S. Zhang, L. Gu, L. Wang, X.-C. Ma, Q.-K. Xue, 2D Mater. **2016**, *3*, 024002.

[11] W. Zhao, M. Li, C.-Z. Chang, J. Jiang, L. Wu, C. Liu, J. S. Moodera, Y. Zhu, M. H. Chan, Sci. Adv. **2018**, 4, eaao2682.

[12] H. Zhang, D. Zhang, X. Lu, C. Liu, G. Zhou, X. Ma, L. Wang, P. Jiang, Q.-K. Xue, X. Bao, Nat. Commun. **2017**, *8*, 214.

[13] G. Khalsa, A. H. MacDonald, Phys. Rev. B **2012**, *86*, 125121.

[14] R. Neville, B. Hoeneisen, C. Mead, J. Appl. Phys. **1972**, *43*, 2124.
